# Ethionamide Preconditioning Enhances the Proliferation and Migration of Human Wharton’s Jelly-Derived Mesenchymal Stem Cells

**DOI:** 10.3390/ijms21197013

**Published:** 2020-09-23

**Authors:** Na-Hee Lee, Su Hyeon Myeong, Hyo Jin Son, Jung Won Hwang, Na Kyung Lee, Jong Wook Chang, Duk L. Na

**Affiliations:** 1 Department of Neurology, Samsung Medical Center, Sungkyunkwan University School of Medicine, 81 Irwon-ro, Gangnam-gu, Seoul 06351, Korea; lnahee@skku.edu (N.-H.L.); soarmsh@skku.edu (S.H.M.); sonhj825@gmail.com (H.J.S.); jung89@skku.edu (J.W.H.); 2 Department of Health Sciences and Technology, SAIHST, Sungkyunkwan University, 81 Irwon-ro, Gangnam-gu, Seoul 06351, Korea; 3 Neuroscience Center, Samsung Medical Center, 81 Irwon-ro, Gangnam-gu, Seoul 06351, Korea; 4 Stem Cell & Regenerative Medicine Institute, Samsung Medical Center, 81 Irwon-ro, Gangnam-gu, Seoul 06351, Korea; nakyunglee@skku.edu; 5 School of Medicine, Sungkyunkwan University, 81 Irwon-ro, Gangnam-gu, Seoul 06351, Korea; 6 Samsung Alzheimer Research Center, Samsung Medical Center, 81 Irwon-ro, Gangnam-gu, Seoul 06351, Korea; 7 R & D Center, ENCell Co. Ltd., Seoul 06072, Korea

**Keywords:** mesenchymal stem cells (MSCs), proliferation, migration, paracrine factors, survival, ethionamide-preconditioned MSCs (ETH-MSCs)

## Abstract

Mesenchymal stem cells (MSCs) are a useful source for cell-based therapy of a variety of immune-mediated diseases, including neurodegenerative disorders. However, poor migration ability and survival rate of MSCs after brain transplantation hinder the therapeutic effects in the disease microenvironment. Therefore, we attempted to use a preconditioning strategy with pharmacological agents to improve the cell proliferation and migration of MSCs. In this study, we identified ethionamide via the screening of a drug library, which enhanced the proliferation of MSCs. Preconditioning with ethionamide promoted the proliferation of Wharton’s jelly-derived MSCs (WJ-MSCs) by activating phosphatidylinositol 3-kinase (PI3K)/Akt and mitogen-activated protein kinase/extracellular signal-regulated protein kinase kinase (MEK)/extracellular signal-regulated kinase (ERK)1/2 signaling. Preconditioning with ethionamide also enhanced the migration ability of MSCs by upregulating expression of genes associated with migration, such as C-X-C motif chemokine receptor 4 (CXCR4) and C-X-C motif chemokine ligand 12 (CXCL12). Furthermore, preconditioning with ethionamide stimulated the secretion of paracrine factors, including neurotrophic and growth factors in MSCs. Compared to naïve MSCs, ethionamide-preconditioned MSCs (ETH-MSCs) were found to survive longer in the brain after transplantation. These results suggested that enhancing the biological process of MSCs induced by ethionamide preconditioning presents itself as a promising strategy for enhancing the effectiveness of MSCs-based therapies.

## 1. Introduction

Stem cells possess self-renewing capabilities and have the potential to differentiate into multiple cell types. Several types of stem cells are used in cell-based therapy such as embryonic stem cells (ESCs), mesenchymal stem cells (MSCs), and induced pluripotent stem cells (iPSCs). Among them, multipotent MSCs are heralded as an effective cell therapy due to the lack of ethical concerns and tumorigenic potential, as well as the ease of procurement, in comparison to other pluripotent stem cells [1]. Moreover, MSCs are considered to be immune-privileged owing to the absence of major histocompatibility complex class II (MHC class II) antigens [2,3]. Human MSCs are isolated from various tissues such as bone marrow [4], umbilical cord blood [5], periodontal ligament [6], adipose tissue [7], and Wharton’s jelly [8].

Previous studies have demonstrated the beneficial effects of MSCs upon injection into the brain. This results in either replacement of damaged cells through trans-differentiation or the treatment of damaged cells via the paracrine action of MSCs [9,10,11]. MSCs are known to exert their therapeutic effects by secreting various cytokines that promote anti-inflammation [12,13], antioxidant activities [14,15], and activation of endogenous neurogenesis [16,17], rather than differentiation into neurons or glial cells. Such therapeutic potential of MSCs can be enhanced upon rapid migration of MSCs to the target site and prolonged survival in the brain to exert these therapeutic effects.

Several studies have proposed strategies to improve biological processes including migration and proliferation of MSCs: preconditioning of MSCs with specific factors [18,19], hypoxic preconditioning [20,21,22], genetic modification [23,24], and encapsulation [25,26]. Preconditioning of MSCs with pharmacological agents can be another effective method as it is relatively simple and less time-consuming, especially when preconditioning with clinically approved drugs. Many studies have reported that the preconditioning of MSCs with pharmacological agents increases their proliferation, migration, and differentiation ability in vitro, and enhances their therapeutic effects on diverse disease models in vivo [27,28,29].

**Figure 1:**
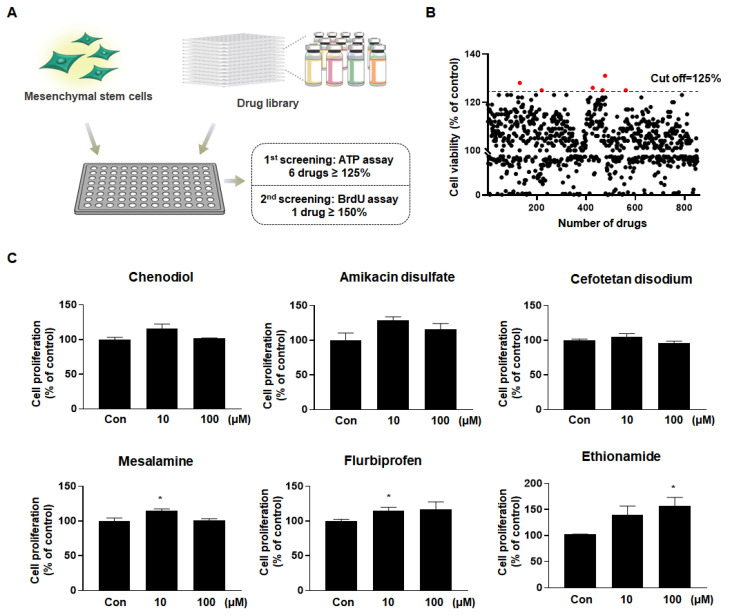
Ethionamide was selected to promote the proliferation of mesenchymal stem cells (MSCs). (**A**) Experimental design of drug library screening. MSCs were exposed to a drug library consisting of 850 FDA-approved compounds, and ethionamide was selected after two screenings. (**B**) MSCs were treated with 10 μM of drugs and the cell viability was measured by ATP assay. Six drugs were selected as a result of the first screening (cut off ≥125%). (**C**) Cell proliferation was measured by BrdU assay after treating MSCs with 10 μM and 100 μM of six drugs. The results obtained from three independent experiments were expressed as a percent of untreated control ± SEM; * *p* < 0.05 vs. untreated control.

After screening a United States Food and Drug Administration (U.S. FDA) approved library consisting of 850 drugs, a preliminary study was conducted on several candidates to evaluate their potential abilities to increase the viability of MSCs. Ethionamide, an antibiotic commonly used to treat tuberculosis, was selected from the secondary screening (Figure 1). Originally, ethionamide is used in clinics worldwide to treat tuberculosis. The known mechanism of tuberculostatic ethionamide is via disruption of mycolic acids, a type of long fatty acids found in bacterial cell wall [30,31]. However, in this study, ethionamide is used for preconditioning of MSCs instead. The aims of this study were (1) to establish an optimal preconditioning condition of ethionamide to maximize the proliferation of MSCs; (2) to investigate whether the ethionamide-preconditioned MSCs (ETH-MSCs) enhance proliferation, migration, and secretion of therapeutic factors in vitro, and (3) whether ETH-MSCs have an increased survival rate in vivo compared to that of naïve MSCs.

## 2. Results

### 2.1. Ethionamide Was Selected for the Preconditioning of MSCs

To find the appropriate drug candidates for the preconditioning of MSCs, a drug library consisting of FDA-approved 850 drugs was purchased and processed in human MSCs (Figure 1A). First, the cell viability was assessed using the ATP assay, which quantified the viable number of cells. Based on our results, six drugs that increased the cell viability (125% or more) were selected (Figure 1B). The details of the selected drugs are described as follows: Chenodiol is a bile acid used to dissolve gallstones; amikacin and cefotetan are antibiotics that exhibit antibacterial efficacy; mesalamine, also known as 5-aminosalicylic acid (5-ASA), is an anti-inflammatory agent used to treat ulcerative colitis; flurbiprofen is a nonsteroidal anti-inflammatory drug (NSAID) used to reduce pain and inflammation; and ethionamide is an antibiotic used to treat tuberculosis. Next, BrdU assay was performed to investigate the effect of the aforementioned drugs on MSCs’ proliferation. While the proliferation of MSCs was increased by less than 1.3-fold with most of the drugs, ethionamide increased cell proliferation by 1.4-fold at 10 μM and 1.6-fold at 100 μM (Figure 1C). According to the results, ethionamide was chosen as a drug to promote the potency of MSCs due to its low cell toxicity and increased cell proliferation in a dose-dependent manner.

### 2.2. Optimum Preconditioning Condition of Ethionamide Was Determined Based on the Concentration and Incubation Period of Ethionamide

**Figure 2:**
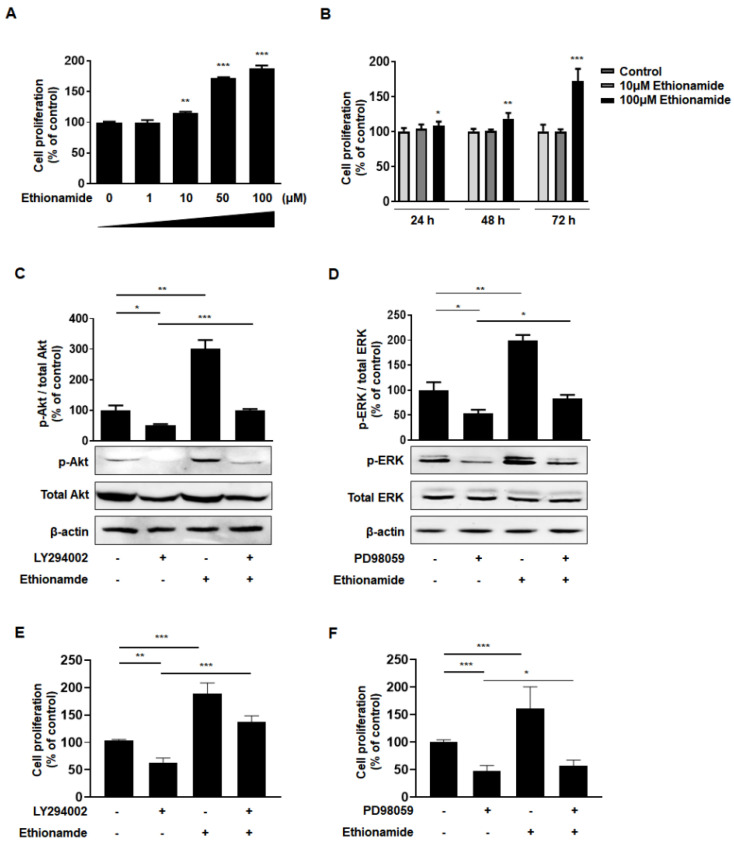
Ethionamide stimulated proliferation of MSCs via activating phosphatidylinositol 3-kinase (PI3K)/Akt and mitogen-activated protein kinase/extracellular signal-regulated protein kinase kinase (MEK/ERK1/2) signaling pathways. (**A**) MSCs were exposed to varying concentrations of ethionamide. The proliferation of MSCs was measured by BrdU ELISA after 72 h incubation. (**B**) The proliferation of MSCs was measured by BrdU ELISA after treatment with 10 μM and 100 μM of ethionamide for 24 h, 48 h, 72 h. (**C**) PI3K/Akt and (**D**) MEK/ERK1/2 signaling pathways were evaluated by Western blotting. The cells were treated with LY294002 (30 μM), the inhibitors of PI3K and PD98059 (50 μM), the inhibitors of MEK to suppress signaling pathways. β-actin was used as an internal control. (**E**,**F**) After the treatment of the cells with ethionamide and inhibitors, the proliferation of MSCs was measured by the BrdU ELISA. The results obtained from three independent experiments were expressed as a percent of untreated control ± SEM; * *p* < 0.05, ** *p* < 0.01 and *** *p* < 0.005 vs. untreated control.

WJ-MSCs were exposed to varying concentrations of ethionamide to assess whether ethionamide affects the proliferation of MSCs. Compared to the untreated control group, the proliferation of ethionamide-treated MSCs was 1.7-fold higher at 50 μM and 1.8-fold higher at 100 μM of ethionamide (Figure 2A). Drug-induced cytotoxicity was observed in MSCs upon treatment with more than 100 μM of ethionamide (data not shown). To set up the optimal conditions for preconditioning, MSCs were treated with 10 μM and 100 μM concentrations of ethionamide at various time points. Compared to the untreated control group, proliferation was increased by 1.1-fold at 24 h, 1.3-fold at 48 h, and 1.7-fold at 72 h after treatment with 100 μM ethionamide (Figure 2B). Based on these results, the optimum concentration of ethionamide and incubation period were determined as 100 μM and 72 h. The characteristics of ethionamide preconditioned MSCs (ETH-MSCs) were investigated to validate their stemness. The expression of surface markers was measured by fluorescence-activated cell sorting (FACS) analysis and more than 95% of the positive markers such as CD44, CD73, CD90, CD105, and CD166 were expressed in both naïve MSCs and ETH-MSCs, whereas less than 1% of the negative markers such as CD11b, CD14, CD19, CD34, CD45, and HLA-DR were expressed both naïve MSCs and ETH-MSCs (Figure S1A). Additionally, ETH-MSCs were able to differentiate into three types of cells, similar to the naïve MSCs (Figure S1B). Collectively, these results showed that ETH-MSCs maintained the representative characteristics of MSCs.

### 2.3. Ethionamide Enhanced the Proliferation of Mscs via Activation of PI3K/Akt and MEK/ERK1/2 Signaling Pathways

We analyzed the molecular mechanism underlying the induction of MSCs’ proliferation caused by ethionamide. PI3K/Akt and MEK/ERK1/2 are well-known signaling pathways involved in cell proliferation via phosphorylation [32,33]. Preconditioning with ethionamide increased the levels of phospho-Akt by 3.0-fold and phospho-ERK1/2 levels by 1.9-fold compared to that in naïve MSCs (Figure 2C,D). These findings were corroborated by results that we obtained from an additional experiment where we treated MSCs with inhibitors. The inhibitors, LY294002 and PD98059, significantly decreased phospho-Akt and phospho-ERK levels by 52% and 53%, respectively. After treatment of inhibitors, ethionamide was also treated, and PI3K/Akt and MEK/ERK1/2 signaling pathways were restored by 100% and 83% in the combined group of ethionamide and inhibitors (Figure 2C,D). This result indicated that ethionamide activated both PI3K/Akt and MEK/ERK1/2 signaling pathways effectively. Due to their roles in signal transduction, the Janus kinase/signal transducers and activators of transcription (JAK/STAT) signaling pathway have been considered as therapeutic targets [34]. Activated JAKs trigger a signaling cascade that leads to the phosphorylation of STATs. Phosphorylated STATs translocate to the nucleus and bind to specific response elements to activate or inhibit the expression of the target genes. Out of the 7 STAT family members, STAT3 promotes cell proliferation, migration, and survival [35]. Therefore, we examined the potential effects of ethionamide preconditioning on the STAT3 signaling pathway. As shown in Figure S2A, a significant difference in the ratio of phospho-STAT3 to STAT3 was not observed between the naïve and ETH-MSCs.

To confirm whether the effect of ethionamide on the signaling pathways was associated with cell proliferation, the level of cell proliferation was assessed after treatment with ethionamide and inhibitors. The results indicated that the proliferation of MSCs was decreased in the group treated with inhibitors. However, the inhibition of cell proliferation was alleviated by ethionamide treatment (Figure 2E,F). Thus, ethionamide-treated MSCs enhanced cell proliferation by activating the PI3K/Akt and MEK/ERK1/2 signaling pathways.

### 2.4. Ethionamide Increased the Migration Ability of Mscs via Expression of Migration-Related Genes

**Figure 3:**
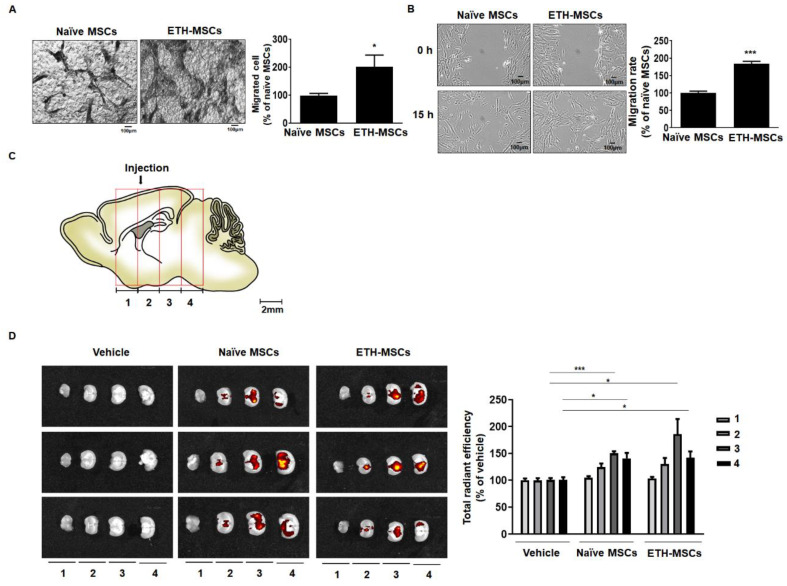
Ethionamide increased the migration ability of MSCs. The migration ability of MSCs was measured by the Boyden chamber assay and wound healing assay in vitro. (**A**) MSCs were cultured in transwell with 0.8 um pore size and after 24 h, migrated cells were stained with hematoxylin. (**B**) A representative image obtained after scratching the surface of 100% confluent MSCs and after 15 h, narrowed gap was measured using Image J. (**C**) The migration ability of MSCs was measured ex vivo. After injection, the mouse brain tissues were divided into four regions. (**D**) The maintenance of MSCs in each region was monitored by optical imaging. Results obtained from three independent experiments are expressed as percent of naïve MSCs or vehicle ± SEM; * *p* < 0.05 and *** *p* < 0.005 vs. naïve MSCs or vehicle control. Vehicle; the media used to resuspend the MSCs for injection. Scale bars = 100 μm.

The effects of ethionamide on the migration ability of MSCs were tested by performing a Boyden chamber assay and wound healing assay in vitro. According to the results of the Boyden chamber assay, the number of cells that migrated towards the lower side of the membrane was increased by 2.0-fold in ETH-MSCs after 24 h (Figure 3A). In the wound healing assay, the migration ability of ETH-MSCs was enhanced to 1.8-fold compared to that of the naïve MSCs (Figure 3B). MSCs were injected into the brain of wild type (WT) mice to further determine the migration ability of MSCs in vivo. The brain samples were harvested after one week and divided into four regions, where the residual MSCs were assessed in each of the four regions (Figure 3C). The injection tract was identified in region two, and most of the residual MSCs were detected in regions three and four. Although the injected ETH-MSCs demonstrated a higher survival rate in the region three compared to that in the regions of naive MSCs, no significant difference was observed between the two groups in other regions (Figure 3D).

**Figure 4:**
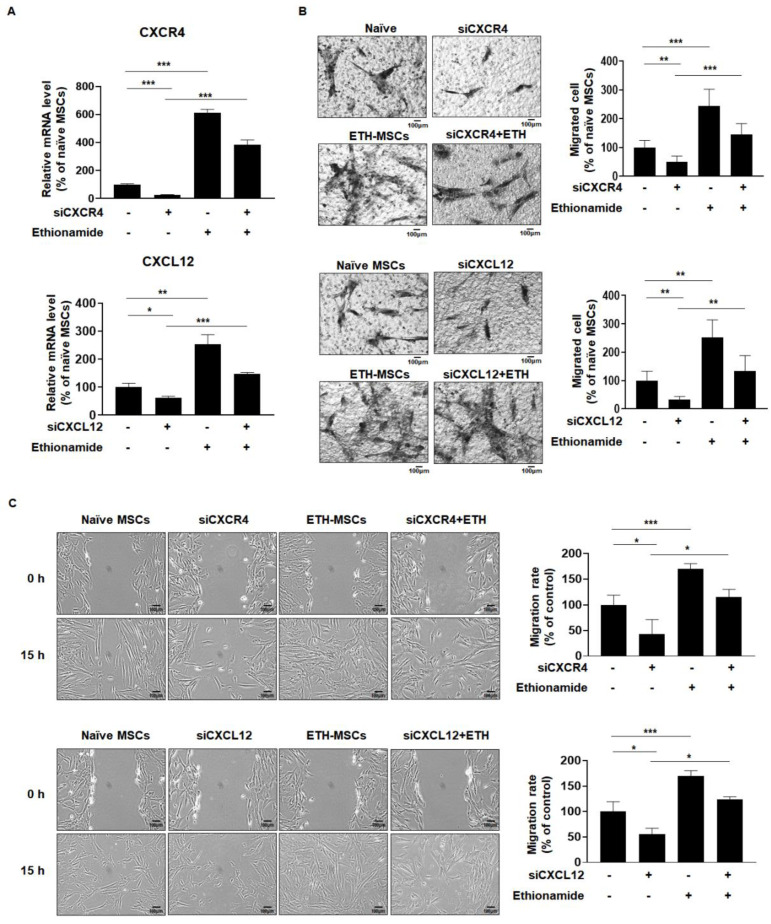
Ethionamide enhanced the migration ability of MSCs via the expression of CXCR4 and CXCL12. (**A**) The effects of siRNAs and ethionamide on CXCR4 and CXCL12 expression were measured by RT-qPCR. The effects of siRNAs and ethionamide on the migration ability were measured by the (**B**) Boyden chamber assay and (**C**) Wound healing assay. The results obtained from three independent experiments are expressed as the percent of untreated control ± SEM; * *p* < 0.05, ** *p* < 0.01 and *** *p* < 0.005 vs. untreated control. Scale bars = 100 μm.

To further elucidate the underlying mechanism of the effect of ethionamide on the migration of MSCs, the levels of C-X-C motif chemokine receptor type 4 (CXCR4) and C-X-C motif chemokine ligand 12 (CXCL12) were measured since they are known as the key regulators of cell migration [36]. In ETH-MSCs, CXCR4 expression levels were increased by 6.0-fold and CXCL12 expression levels were increased by 2.5-fold compared to naive MSCs (Figure 4A). To determine whether both CXCR4 and CXCL12 were involved in the migration of ETH-MSCs, individual-specific small interfering RNAs (siRNAs) were transfected into MSCs. RT-qPCR results validated that the expression of CXCR4 and CXCL12 was reduced by 50% in the siRNA -treated group, whereas the CXCR4 expression was increased to approximately 3.8-fold and CXCL12 expression was increased to approximately 1.5-fold upon co-treatment with ethionamide (Figure 4A). According to the results of the Boyden chamber assay and wound healing assay, the migration of MSCs was initially blocked by siRNA of CXCR4 and CXCL12. However, ethionamide attenuated the effects of siRNAs and restored the migration ability of MSCs (Figure 4B,C). Taken together, these data indicated that the ethionamide-mediated stimulation of endogenous CXCR4 and CXCL12 is critical for MSCs to improve their migration ability.

### 2.5. Ethionamide Increased the Secretion of Paracrine Factors

Human MSCs are known to release several paracrine factors that affect the disease microenvironment. In particular, Wharton’s jelly-derived MSCs tend to secrete factors related to angiogenesis and neurogenesis [37]. Thus, we investigated the levels of several neurotrophic and growth factors associated with angiogenesis and neurogenesis in the brain. Previous studies have shown that MSCs secrete brain-derived neurotrophic factor (BDNF) for neuronal protection, making it a promising candidate for the treatment of neurodegenerative diseases [38,39]. Compared to naive MSCs, the level of BDNF was 1.3-fold higher in ETH-MSCs (Figure S3A). Previous findings also suggested that growth factors such as vascular endothelial growth factor (VEGF), insulin-like growth factor (IGF-1), and hepatocyte growth factor (HGF) are also involved in angiogenesis and neuroprotection [40,41,42]. Overexpression of VEGF enhances the survival of human neural stem cells (NSCs) and leads to behavioral improvement in mouse stroke models [42]. Human MSCs improved functional recovery and increased neurogenesis in the brain by inducing the expression of IGF-1 [41]. HGF is a potent neurotrophic factor involved in the development of the nervous system and exhibits a therapeutic effect in various neurodegenerative diseases [40]. In accordance with these findings, experiments were performed to assess the levels of VEGF, IGF-1, and HGF and were significantly increased in ETH-MSCs, with a 1.7-, 1.3-, and 1.8-fold increase at 100 µM, respectively. (Figure S3B–D). Taken together, preconditioning of MSCs with ethionamide was able to induce the secretion of paracrine factors, thereby highlighting the potential of ETH-MSCs for application in cell-based therapeutics.

### 2.6. Ethionamide Enhanced the Survival of Mscs In Vivo

**Figure 5:**
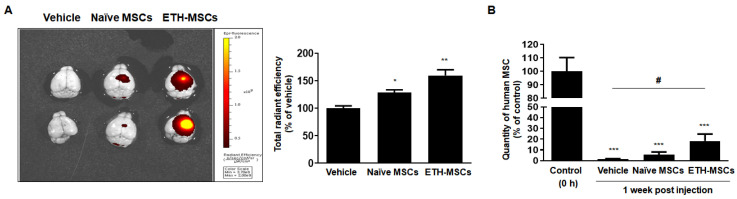
Preconditioning with ethionamide enhanced the survival of MSCs in vivo. The survival rate of MSCs was assessed. (**A**) MSCs were shown by red fluorescence signal of optical imaging ex vivo. (**B**) The quantification of survived MSCs was investigated by qPCR using human-specific Alu primer. Results obtained from three independent experiments are expressed as percentage of control ± SEM; * *p* < 0.05, ** *p* < 0.01, and *** *p* < 0.005 vs. control (0 h). Control (0 h); the brain tissue extracted immediately after MSCs injection. # *p* < 0.05 vs. vehicle. Vehicle; the media used to resuspend the MSCs for injection.

Preconditioning of MSCs with ethionamide increased the cell migration ability and secretion of paracrine factors. Identifying ways to prolong the survival of MSCs after transplantation is crucial to exert their therapeutic effects. Residing MSCs were assessed by the detection of MSCs that had been tagged with fluorescent dye before injecting into the wildtype (WT) mouse brains. One week after injection, the mice were sacrificed and MSCs in the brain were detected by optical imaging ex vivo. As shown in Figure 5A, the remaining number of MSCs was higher in the ETH-MSCs group than that in the naive MSCs group seven days post-administration. Assessed via Alu qPCR, compared to the naïve MSCs group, the number of residual MSCs in the brain was 1.2-fold higher for the ETH-MSCs group. Compared to the positive control group where the brain tissues were harvested immediately after MSC injection, only 5.7% and 18% of naïve MSCs and ETH-MSCs were detected in the mouse brain, respectively. Thus, the number of residual MSCs for the ETH-MSCs group was 3.2-fold higher when compared to that of naïve MSCs. Based on the results, we confirmed that ETH-MSCs had a higher survival rate than naïve MSCs after brain transplantation.

## 3. Discussion

Human MSCs are widely used as a potential remedy for incurable diseases such as Parkinson’s disease, amyotrophic lateral sclerosis, Huntington’s disease, and Alzheimer’s disease, possibly through secretion of neurotrophic factors and promotion of neurogenesis [38,43,44,45]. Studies that have evaluated stem cell-based therapy have demonstrated that the properties of MSCs are influenced by the disease microenvironment. MSCs transplanted in animal disease models are exposed to harsh environments, which can affect their survival and reduce their migratory abilities [46]. This can eventually exert detrimental effects on the therapeutic benefits of MSCs. Therefore, developing strategies to overcome such obstacles is necessary. In the present study, we demonstrated that the preconditioning of MSCs with ethionamide resulted in increased cell proliferation and migration, as well as enhanced paracrine potency. Collectively, these enhancements led to improved therapeutic effects and increased the survival of MSCs in vivo. To the best of our knowledge, this is the first study to evaluate the ability of ethionamide to increase the cellular functions of human MSCs.

Our study investigated the P13k/Akt and MEK/ERK1/2 signaling pathways to study the mechanisms underlying the increase in proliferation of MSCs in vitro upon treatment with ethionamide. PI3K/Akt and MEK/ERK1/2 signaling pathways have anti-apoptotic effects [47]. Previous studies have shown that the proliferation of MSCs is increased via upregulation of PI3K/Akt and MEK/ERK1/2 signaling pathways [48,49,50]. As expected, ethionamide activated PI3K/Akt and MEK/ERK1/2 signaling pathways by increasing phosphorylation as well as attenuating the inhibitory effects of PI3K and MEK (Figure 1). The level of proliferation was markedly lower in the MSCs treated with MEK/ERK1/2 inhibitors compared to that in the group treated with PI3K/Akt inhibitors, and the restoring effect of ethionamide was also lower in the MEK/ERK1/2 pathway. These results suggested that the PI3K/Akt pathway is the underlying signaling pathway mediating the proliferative efficacy of ETH-MSCs.

The therapeutic potential of MSCs is attributed to their migration ability as well as their proliferative capacities. It is necessary for MSCs to migrate to the damaged tissue in order to exert their therapeutic efficacy. Upon an increase in CXCL12 expression in inflamed tissues, MSCs detect these changes via expression of CXCR4, a receptor for CXCL12, and migrate to the injured tissue. Similar observations previously noted that CXCL12 expression promotes rapid migration to damaged tissues such as the brain, heart, and bones [51,52]. Preconditioning with ethionamide enhanced the migration ability of MSCs, and CXCR4 and CXCL12 were involved in this process (Figure 4). In previous studies, the administration route and dosage were optimized to acquire even distribution of the transplanted MSCs in both canine and mouse models [53,54]. Based on these findings, MSCs were injected into the lateral ventricle of the mouse brain and ETH-MSCs demonstrated a consistently higher migration ability compared to that of naive MSCs in vivo.

In addition to their proliferation and migration abilities, ETH-MSCs also possessed the ability to produce paracrine factors that are beneficial in neurodegenerative diseases. Mechanisms underlying these effects remain to be elucidated, but PI3K/Akt and MEK/ERK1/2 signaling pathways are known to be involved in not only cell proliferation and migration but also in neuroprotective effects by regulating the secretion of paracrine factors. Previous reports suggested that PI3K/Akt and MEK/ERK1/2 signaling pathways regulate transcription activities including neurotrophic factors. The MEK/ERK1/2 pathway phosphorylates the transcription factor cAMP response element binding protein (CREB), which translocates into the nucleus and induces the transcription of BDNF and IGF-1 [55,56,57,58]. Recent studies have demonstrated that AKT and ERK1/2 also stimulate VEGF expression via HIF-1α activation [59] and that HGF production is upregulated by the RhoA-PI3K/Akt-MEK/ERK1/2 signaling axis [60,61]. Alongside these findings, the expression of neurotrophic and growth factors such as BDNF, VEGF, IGF-1, and HGF was increased in ETH-MSCs (Figure S3). The aforementioned results highlight the therapeutic potential of ETH-MSCs. Thus, it can be assumed that ETH-MSCs induce secretion of paracrine factors via activation of PI3K/Akt and MEK/ERK1/2 pathways. However, further studies are required to support the proposed mechanism.

Many studies have reported on various preconditioning methods to enhance the survival of MSCs [62,63,64]. However, few studies have explored how the survival of these preconditioned MSCs are altered following transplantation into the brain. In particular, most human MSCs transplanted into the mouse brain are barely detectable by day 7 post-transplantation [39,53,65]. As shown in Figure 5, the survival of ETH-MSCs was greater than that of naïve MSCs in the mouse brain. However, the amount of ETH-MSCs remaining after one week was less than expected. This may have been possibly associated with immune reactions generated by the host following transplantation of xenogeneic MSCs [66,67]. Once the issue arising from an immune response can be overcome, enhanced survival rate of ETH-MSCs could be effective in a myriad of neurological disorders such as stroke, traumatic brain injury as well as neurodegenerative disorders such as Alzheimer’s disease and Parkinson’s disease.

The known side effects of ethionamide include nausea and liver inflammation [68]. However, the possibility of occurrence of those side effects when injected into the human brain is precluded since preconditioning of MSCs with ethionamide is used to increase the biological capacity of MSCs rather than being administered directly into the body. In addition, there was hardly any residual amount of ethionamide left in ETH-MSCs after sufficient washing procedures as in the standard operating procedures. Mortality or signs of abnormality were not observed from the mice that received transplantations of ETH-MSCs, which further support the safety of ethionamide preconditioning. The fact that ethionamide is a stable FDA-approved drug is a major advantage with respect to its clinical translation. It is also possible to simplify the process of clinical trials as the drug has already been commercialized. Our findings may help enhance the effectiveness of stem cell research and its therapeutic application in various other diseases.

## 4. Materials and Methods

### 4.1. Ethical Statement

This study was approved by the Institutional Animal Care and Use Committee (IACUC) (20170317001) (6 April 2017) of the Samsung Biomedical Research Institute (SBRI) at Samsung Medical Center (SMC), Republic of Korea. As an accredited facility of the Association for Assessment and Accreditation of Laboratory Animal Care International (AAALAC International), the SBRI acts in accordance with the guidelines set forth by the Institute of Laboratory Animal Resources (ILAR). The study was also approved by the Institutional Review Board (IRB No. 2016-07-102) of SMC after informed consent was received by all of the patients undergoing childbirth. Human WJ-MSCs were isolated and provided according to the standard operating procedures of the Good Manufacturing Practice (GMP) facility at SMC.

### 4.2. Cell Culture and Treatment

Human WJ-MSCs were cultured in 1× minimal essential medium alpha (MEMα, Gibco-Thermo Fisher Scientific, Waltham, MA, USA) supplemented with 10% fetal bovine serum (FBS, Biowest, Riverside, MO, USA) and 0.5% gentamicin (Gibco-Thermo Fisher Scientific, Waltham, MA, USA). For preconditioning, MSCs were seeded in a 75T flask (2.3 × 10^6^ cells) in MEMα with 3% FBS and 0.5% gentamicin and the cells were treated with 100 μM of ethionamide (Enzo Life Science, Farmingdale, NY, USA). The cells were maintained at 37 °C for 72 h in a humidified incubator with 95% air and 5% CO_2_. To inhibit PI3K/Akt and MEK/ERK1/2 signaling pathways, LY294002 (30 μM, Cell Signaling Technology, Danvers, MA, USA), which is an inhibitor of the PI3K/Akt pathway and PD98059 (50 μM, Cell Signaling Technology), which is an inhibitor of the MEK/ERK1/2 pathway, were used. The cells were treated with the inhibitors for 1 h followed by ethionamide (100 μM) treatment and maintained at 37 °C for 72 h in a humidified incubator.

### 4.3. Measurement of Cell Viability and Proliferation

To screen the FDA-approved drug library (Enzo Life Sciences Inc., Farmingdale, NY, USA), MSCs were seeded (1.0 × 10^4^ cells per well) onto a 96-well plate and treated with 10 µM of drug library. After 72 h of incubation, the CellTiter-Glo^®^ Luminescent Cell Viability Assay kit (Promega, Madison, WI, USA) was used, according to the manufacturer’s instructions to investigate the change in viability. For secondary screening, MSCs were seeded (1.0 × 10^4^ cells per well) onto a 96-well plate and were treated with 10 μM and 100 μM of 6 drug candidates. After 72 h of incubation, a 5-bromo-2′-deoxyuridine (BrdU) incorporation enzyme-linked immunosorbent assay (ELISA), (Roche, Basel, Switzerland) was performed according to the manufacturer’s instructions. To investigate the optimal concentration of ethionamide, MSCs were treated with varying concentrations of ethionamide, and the BrdU ELISA was performed as described above.

### 4.4. Fluorescence-Activated Cell Sorting (FACS) Analysis

In order to confirm the characteristics of MSCs, the surface antigen expression of MSCs was confirmed by using a FACS analysis. The cells (2.0 × 10^5^) were stained with followed antibodies: CD44, CD73, CD90, CD105, CD166, CD11b, CD14, CD19, CD34, CD45 and HLA-DR (BD bioscience, San Jose, CA, USA). The markers expression was quantified by using the MACSQuant^®^ Analyzer (Miltenyi Biotec, San Diego, CA, USA).

### 4.5. Differentiation of MSCs

To induce differentiation into osteoblast and adipocyte, MSCs were seeded (1.0 × 10^5^ per well) onto a 6-well plate. For 3 weeks, media were changed twice a week by using an adipogenesis differentiation kit and osteogenesis differentiation kit (Gibco-Thermo Fisher Scientific). For chondrogenesis, MSCs (5.0 × 10^5^) were cultured in chondrogenic induction media: high glucose DMEM, 100 μM dexamethasone (Sigma Aldrich, St. Louis, MO, USA) and 50 mg/mL L-ascorbic acid (Sigma-Aldrich), 100 ng/mL sodium pyruvate (Sigma-Aldrich), 40 mg/mL L-proline (Sigma-Aldrich), 10 ng/mL transforming growth factor (TGFβ-3; R & D systems, Minneapolis, MN, USA), 500 ng/mL bone morphogenetic protein-6 (BMP-6; R & D systems) and 50 mg/mL ITS+ premix (Becton Dickinson, Franklin Lakes, NJ, USA) for 4 weeks. To confirm the differentiation of adipocytes, the cells were fixed with 4% paraformaldehyde (PFA) and stained with Oil red O (Sigma Aldrich). Osteoblast differentiation was confirmed by alkaline phosphatase (Sigma Aldrich) staining. In the case of chondrocytes, the cells were fixed with Tissue-Tek^®^ O.C.T.™ Compound (Sakura Europe, Flemingweg, Netherland) and cut to a thickness of 10 μm slides and stained with 0.1% safranin-O (Biosesang, Sungnam, Republic of Korea) solution. The quantification of adipocytes was performed by adding 100% isopropanol and absorbance were measured at 500 nm. Osteoblast quantification was performed using an alizarin red s staining assay kit. Chondrocyte was quantified using the image J program.

### 4.6. Western Blotting

To confirm the effects of ethionamide on signaling pathways, MSCs were harvested after preconditioning and lysed using radioimmunoprecipitation (RIPA) buffer. Equal amounts (20 μg) of protein were separated on 10% sodium dodecyl sulfate (SDS) polyacrylamide gel and transferred onto a nitrocellulose membrane. After treatment with blocking solution (5% skim milk, 10 mM Tris-HCl, pH 7.5, 150 mM NaCl and 0.4% Tween-20) for 1 h at room temperature, the membrane was incubated overnight with the appropriate antibodies [Phospho-Akt (1:1000), Total Akt (1:1000), Phospho-ERK1/2 (1:1000), Total ERK1/2 (1:1000), Phospho-STAT3 (1:1000), Total STAT3 (1:1000)] (Cell Signaling Technology) and β-actin (1:5000, Santacruz Biotechnology) at 4 °C, followed by horseradish peroxidase-conjugated secondary antibodies for 3 h at room temperature. Protein bands were then detected by chemiluminescence. Densitometry was performed using the ImageJ program, and the results were normalized against β-actin.

### 4.7. Measurement of Migration In Vitro

Boyden chamber and wound healing assay were performed to investigate the migration ability of MSCs. After 72 h of preconditioning, MSCs were seeded (5.0 × 10^4^ cells per transwell) onto the upper chamber of a transwell insert in serum-free media. Media containing 10% FBS was maintained in the lower chamber. After 24 h, the upper surface of the transwell membranes was swabbed by using a cotton swab. The cells that migrated towards the lower surface of the membranes were fixed with 4% paraformaldehyde (PFA) for 10 min and stained with hematoxylin (Dako-Agilent, Santa Clara, CA, USA). The number of cells was counted in randomly selected fields. For the wound healing assay, MSCs were seeded (2.0 × 10^5^ cells per well) onto a 6-well plate and treated with ethionamide in culture medium containing 3% FBS. Once the cells reached 100% confluence, they were treated with 5μg/mL of mitomycin C (Sigma Aldrich, St. Louis, MO, USA) for 2 h. A sterile pipette tip was used to scratch the bottom of the well and the culture media was changed to media containing 10% FBS. The migration of MSCs into the wound area was evaluated by using the ImageJ program.

### 4.8. RNA Extraction and Reverse Transcription-Quantitative Polymerase Chain Reaction (RT-qPCR)

To investigate gene expression, total RNA was isolated from the cells using TRIzol reagent (Ambion-Thermo Fisher Scientific). Complementary DNA (cDNA) was synthesized using the superscript III cDNA synthesis kit (Invitrogen-Thermo Fisher Scientific). RT-qPCR was performed by mixing the cDNA with PCR mixtures containing SYBR green master mix (Applied Biosystems, CA, USA). The samples used to perform qPCR were subjected to a 40-cycle amplification at 94 °C for 30 s, 60 °C for 40 s, and 72 °C for 1 min. The following primer sequences were used: CXCR4 (forward, GAGTCGATGCTGATCCCAAT; reverse, AAGGCTATCAGAAGCGCAAG); CXCL12 (forward, TCTCAACACTCCAAACTGTGC; reverse, CTTTAGCTTCGGGTCAATGC); BDNF (forward, AGGCTTGACATCATTGGCTGA; reverse, CGTGTACAAGTCTGCGTCCT); VEGF (forward, GGCCAGCACATAGGAGAGATG; reverse, AGGCCCACAGGGATTTTCTT); IGF-1 (forward, TGCTTCCGGAGCTGTGATCTA; reverse, GCTGACTTGGCAGGCTTGA); HGF (forward, GCCCTATTTCTCGTTGTGAAGGT; reverse, CTGTATCTCAAACTAACCATCCATCCTATG); GAPDH (forward, AGGGCTGCTTTTAACTCTGGT; reverse, CCCCACTTGATTTTGGAGGGA). The results were normalized against human GAPDH levels and quantified using the threshold cycle (∆∆Ct).

### 4.9. Small Interfering RNA

Small interfering RNAs (siRNAs) were used to confirm the knockdown effects of genes associated with migration in MSCs. After seeding MSCs (0.5 × 10^5^ cells per well) onto a 6-well plate, the predesigned siRNAs for CXCR4 and CXCL12 (Bioneer, Daejeon, South Korea) were mixed with Lipofectamine RNAiMAX reagent (Invitrogen-Thermo Fisher Scientific) and transfected into the MSCs. After 1 h of transfection, ethionamide (100 μM) was added and the cells were maintained at 37 °C for 72 h.

### 4.10. Quantification of Mscs Migration and Survival In Vivo

To visualize the migration ability and survival rate of MSCs, Vybrant™ DiD cell-labeling solution (Thermo Fisher Scientific) was used according to the manufacturer’s instructions. C57BL/6 mice (Jackson Laboratories, Bar Harbor, ME, USA) were used to investigate the migration and survival of MSCs in vivo. There were three experimental groups: sham (*n* = 5), naïve MSCs (*n* = 5), and ETH-MSCs (*n* = 5). Before surgery, all experimental animals were anesthetized using 5% isoflurane and the anesthesia was maintained with 2% isoflurane during the surgical procedure. Mice were placed on a rodent universal surgical stereotactic frame (Harvard apparatus, Holliston, MA, USA), and their heads were fastened using ear bars. A total of 1.0 × 10^5^ MSCs were suspended in 7 μL of phenol red-free MEMα 1× (Gibco-Invitrogen) and injected into the right lateral ventricle at the following coordinates: M/L = +1.21 mm, A/P = −0.46 mm, D/V = −2.3 mm. Optical imaging (IVIS Spectrum In Vivo Imaging System, Perkin Elmer, Waltham, MA, USA) was performed after one week following the injection. Whole brain samples were harvested and divided into four regions as described previously [53] to investigate the migratory abilities of MSCs. The survival of injected MSCs was confirmed by Alu-qPCR seven days post- injection. The harvested brain samples were homogenized using a lysis buffer and genomic DNA (gDNA) was isolated by using Gentra Puregene Tissue Kit (QIAGEN, Hilden, Germany). Alu qPCR was performed using a mixture containing SYBR green master mix (Applied Biosystems, Foster City, CA, USA), primers for human-specific Alu elements (forward, GGTGAAACCCCGTCTCTACT; reverse, GGTTCAAGCGATTCTCCTGC) and 20 ng of gDNA. Genomic DNA extracted from varying number of human MSCs (10^2^, 10^3^, 10^4^, 10^5^, and 10^6^ cells) was used to create a standard curve. The residing MSCs in the harvested brain samples were quantified by fitting the respective C_T_ values to the standard curve. qPCR was performed for 40 cycles at 95 °C for 15 s, 56 °C for 30 s, and 72 °C for 30 min.

### 4.11. Data Analysis

Data are expressed as the average ± SEM of independent experiments. The results were compared by t-test or one-way analysis of variance (ANOVA) using Prism v8.0 (GraphPad Software). A *p*-value of <0.05 was considered statistically significant for all analyses.

## 5. Conclusions

We demonstrated that the preconditioning MSCs with ethionamide improves proliferation and migration ability in vitro in comparison to naïve MSCs. In addition, it was observed that the survival and migration ability of preconditioned MSCs increased in vivo compared to that of the naïve MSCs. In addition, their ability to increase the secretion of paracrine factors was also demonstrated. Hence, the results of our study suggest that ETH-MSCs have enhanced therapeutic properties via the secretion of beneficial paracrine factors, with prolonged survival rate and migration capacity in various disease models, especially in neurodegenerative diseases.

## Supplementary Materials

Supplementary Materials can be found at https://www.mdpi.com/1422-0067/21/19/7013/s1. Figure S1: Characterization of ETH-MSCs; Figure S2: Ethionamide did not stimulate JAK/STAT signaling pathway; Figure S3: Ethionamide promoted the secretion of paracrine factors.
