# Supplementary material for: Ethionamide Preconditioning Enhances the Proliferation and Migration of Human Wharton’s Jelly-Derived Mesenchymal Stem Cells
Source: Int J Mol Sci. 2020 Sep 23;21(19):7013. doi: 10.3390/ijms21197013 (PMC7583833; doi:10.3390/ijms21197013)
Supplement: Supplementary file 1 [file ijms-21-07013-s001.pdf]

International Journal of *Molecular Sciences*

MDPI

*Supplementary Materials*

# Ethionamide Preconditioning Enhances the Proliferation and Migration of Human Wharton's Jelly-derived Mesenchymal Stem Cells

**Na-Hee Lee [1,2,3,4,†], Su Hyeon Myeong [1,2,3,4,†], Hyo Jin Son [1,3,4,5,†], Jung Won Hwang [1,2,3,4], Na Kyung Lee [4,5,6], Jong Wook Chang [4,7,*] and Duk L. Na [1,2,3,4,6,*]**

1. Department of Neurology, Samsung Medical Center, Sungkyunkwan University School of Medicine, 81 Irwon-ro, Gangnam-gu, Seoul 06351, Korea; lnahee@skku.edu (N.-H.L.); soarmsh@skku.edu (S.H.M.); sonhj825@gmail.com (H.J.S.); jung89@skku.edu (J.W.H.)
2. Department of Health Sciences and Technology, SAIHST, Sungkyunkwan University, 81 Irwon-ro, Gangnam-gu, Seoul 06351, Korea
3. Neuroscience Center, Samsung Medical Center, 81 Irwon-ro, Gangnam-gu, Seoul 06351, Korea
4. Stem Cell & Regenerative Medicine Institute, Samsung Medical Center, 81 Irwon-ro, Gangnam-gu, Seoul 06351, Korea; nakyunglee@skku.edu
5. School of Medicine, Sungkyunkwan University, 81 Irwon-ro, Gangnam-gu, Seoul 06351, Korea
6. Samsung Alzheimer Research Center, Samsung Medical Center, 81 Irwon-ro, Gangnam-gu, Seoul 06351, Korea
7. R&D Center, ENCell Co.Ltd, Seoul 06072, Korea

* Correspondence: jongwook.chang@samsung.com (J.W.C.); dukna@naver.com or dukna@skku.edu (D.L.N.)

† These authors contributed equally to this work.

Received: 21 August 2020; Accepted: 21 September 2020; Published: 23 September 2020

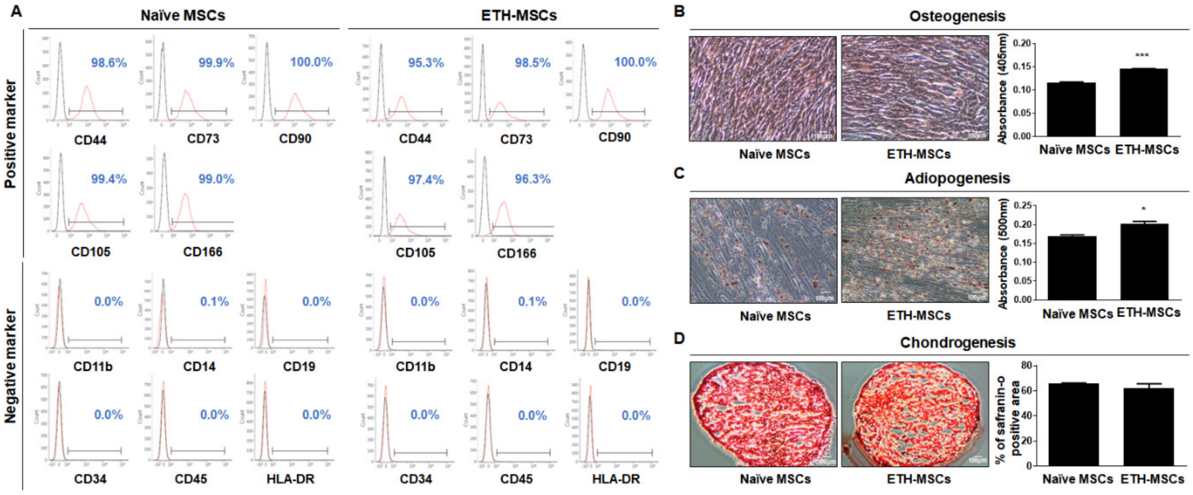


**Figure 1.** Characterization of ETH-MSCs. MSCs characterization was investigated by FACS analysis and differentiation. (A) After 72 h of preconditioning with ethionamide, the surface markers' alteration change of MSCs were confirmed by flow cytometer. (B) Differentiation ability into osteoblast, adipocyte, and chondrocyte was confirmed by the appropriate staining method for each cell type. The results are obtained from three independent experiments are expressed as percent of naive MSCs ± SEM; *$p < 0.05$ and ***$p < 0.005$ vs. naïve MSCs.

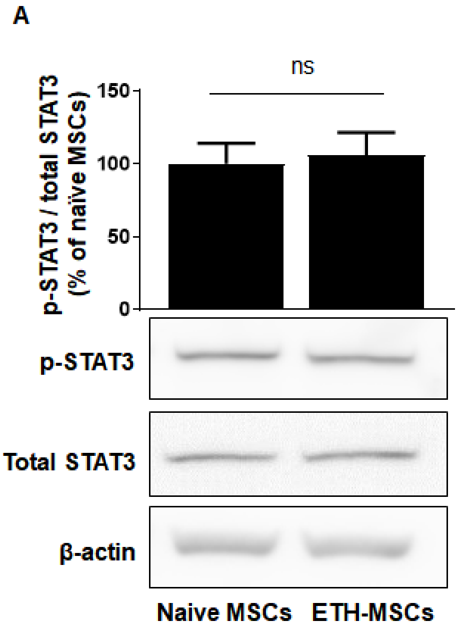


**Figure 2.** Ethionamide did not stimulate JAK/STAT signaling pathway. Ethionamide did not stimulated MSCs' proliferation via activation of STAT3 signaling pathway. (A) MSCs were exposed to 100 µM of ethionamide for 72h. STAT3 signaling pathways were evaluated by western blotting. β-actin was used as an internal control. The results obtained from three independent experiments were expressed as a percent of untreated control ± SEM; ns, not significant.

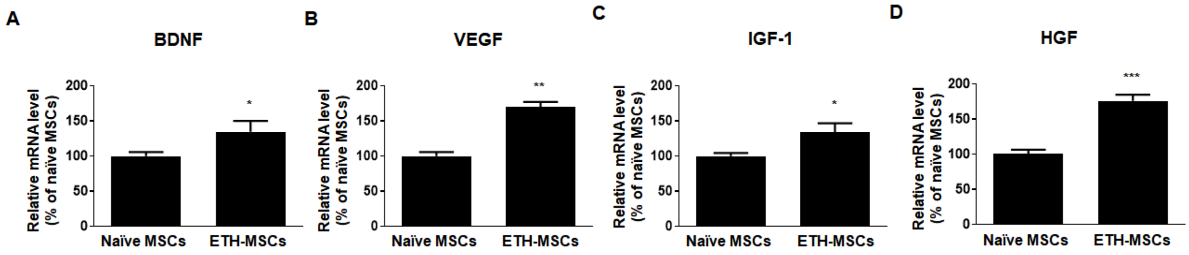


**Figure 3.** Ethionamide promoted the secretion of paracrine factors. After 72 h of preconditioning, the cells were harvested and the total RNA was subjected to RT-qPCR analyses for paracrine factors. GAPDH was used as an internal control. (A) BDNF, (B) VEGF, (C) IGF-1, and (D) HGF were detected in the MSCs. The results obtained from three independent experiments are expressed as percent of naïve MSCs ± SEM; *$p < 0.05$, **$p < 0.01$ and ***$p < 0.005$ vs. naïve MSCs.

© 2020 by the authors. Licensee MDPI, Basel, Switzerland. This article is an open access article distributed under the terms and conditions of the Creative Commons Attribution (CC BY) license (http://creativecommons.org/licenses/by/4.0/).
